# Supplementary figures and images for: Temperature-robust activity patterns arise from coordinated axonal Sodium channel properties
Source: PLoS Comput Biol. 2020 Jul 27;16(7):e1008057. doi: 10.1371/journal.pcbi.1008057 (PMC7410338; doi:10.1371/journal.pcbi.1008057)

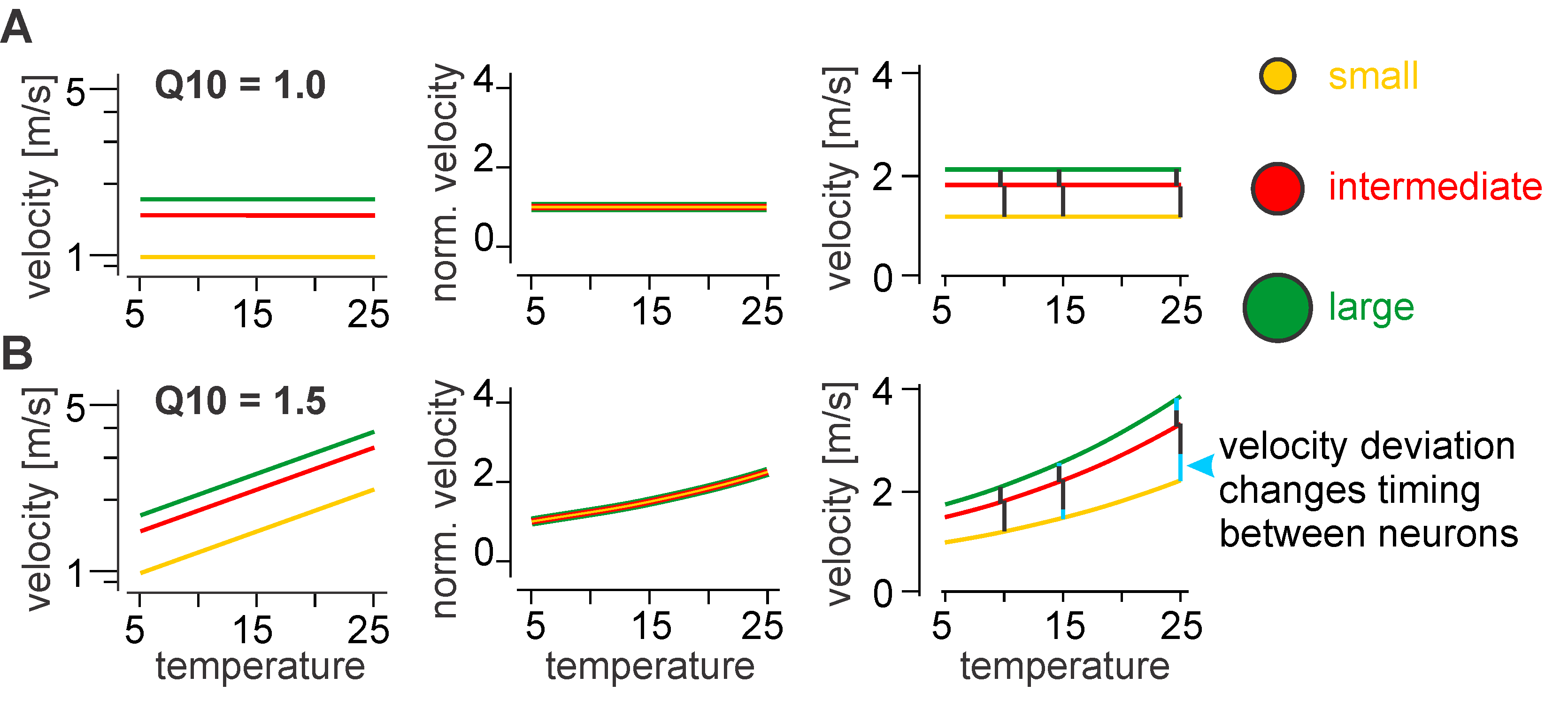

Supplement: S1 Fig — At velocity Q10s greater than one, the difference in velocity between neurons changes with temperature even when all axons have the same Q10. (A) With Q10s of one, the velocities of three different diameter axons are equally insensitive to temperature changes (left, middle). Thus, they show identical velocity differences at all temperatures (right, black bars). Colors denote different axon diameters. (B) At Q10s larger than one, the velocities of the three axons still change proportionately (left, middle), but the difference in velocity between neurons increases (right, blue bars). (TIF) [file pcbi.1008057.s001.tif]

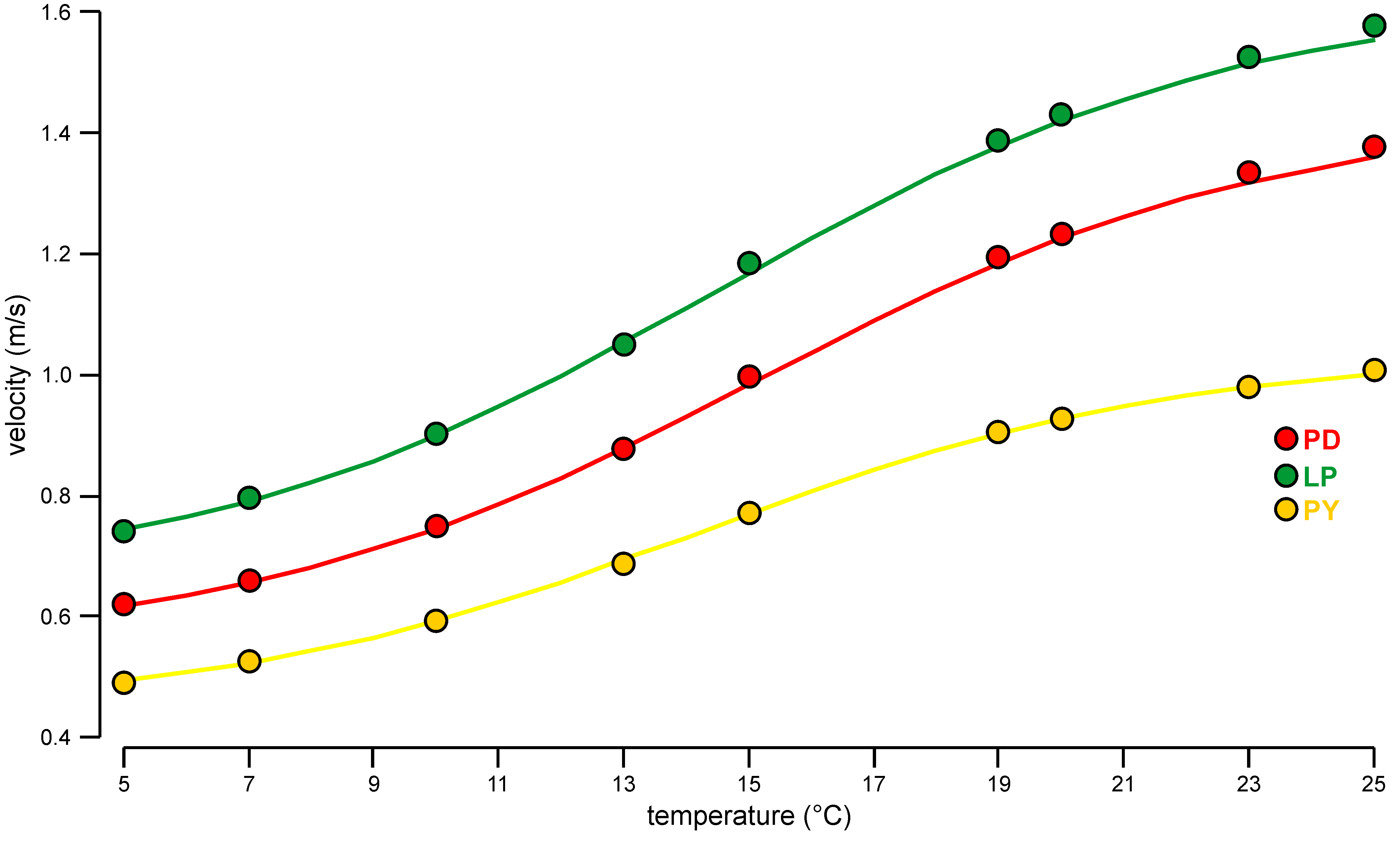

Supplement: S2 Fig — Velocity is plotted and sigmoidal fits for the pyloric axons are given by the equations, PD (red): velocityPD=−0.260+0.4171+e−(Temperature−13.172)4.186, PY (yellow): velocityPY=−0.355+0.3701+e−(Temperature−12.519)3.963, and LP (green): velocityLP=−0.189+0.4011+e−(Temperature−12.561)4.371. (TIF) [file pcbi.1008057.s002.tif]

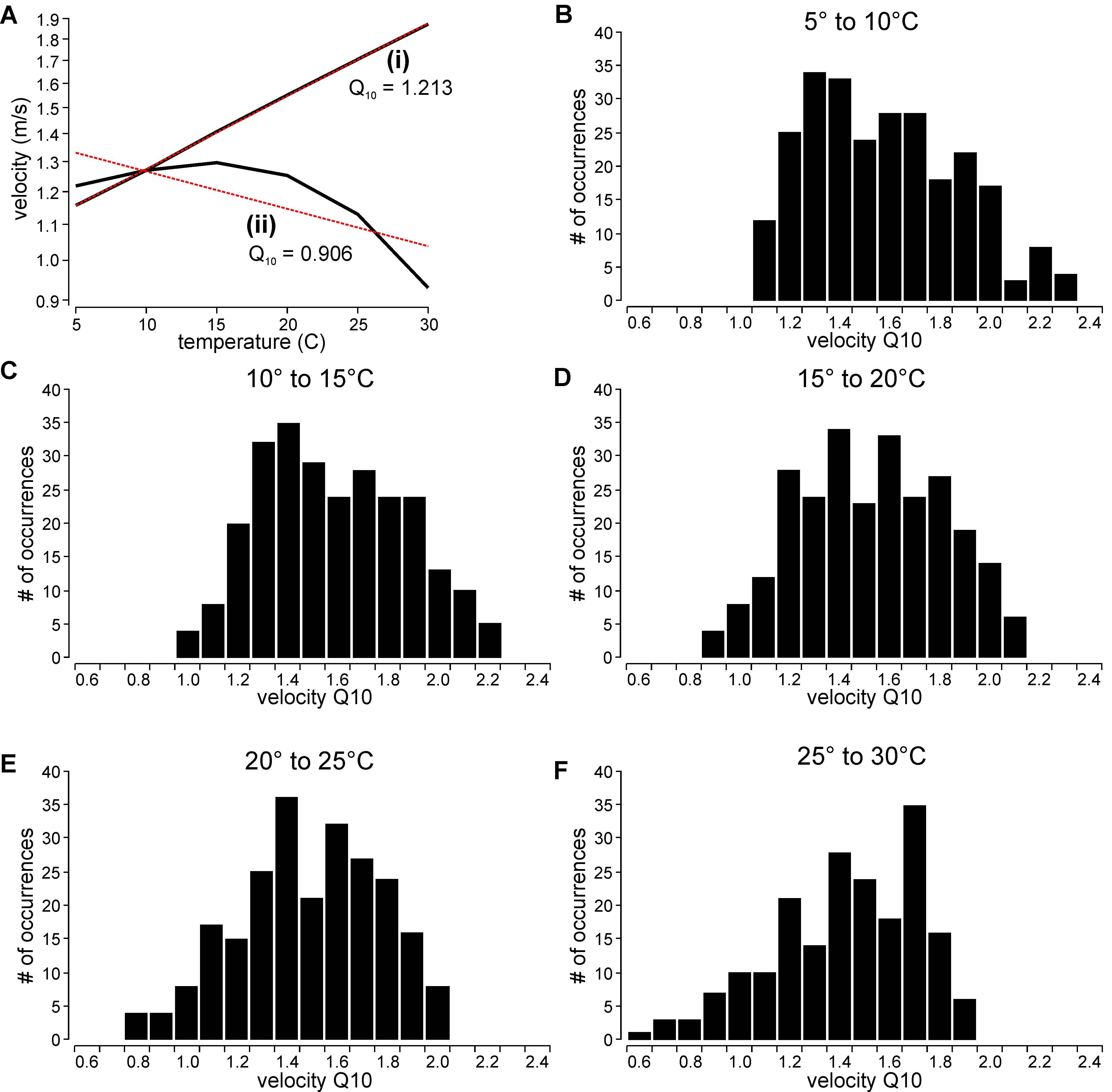

Supplement: S3 Fig — (A) The velocities of two example neurons are plotted on a logarithmic scale to show when a single Q10 value fits well (i) and when it does not fit well (ii). Q10 values are calculated based on the linear regression of the entire temperature range. (B-F) The distribution of velocity Q10s measured in five degree Celsius increments shows a small range in Q10 values with a majority lying within a range of 1.2–1.8. (TIF) [file pcbi.1008057.s003.tif]

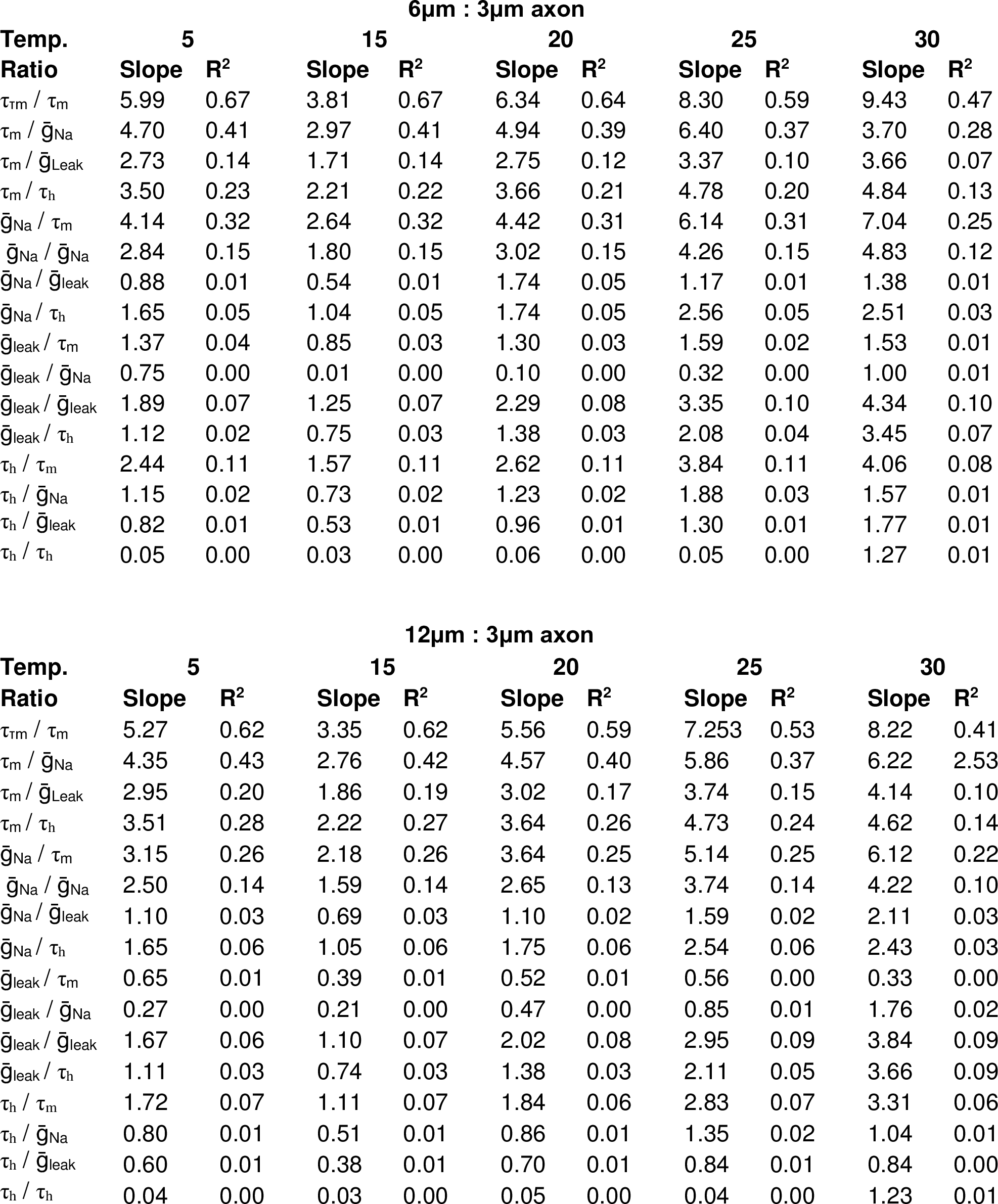

Supplement: S1 Table — A linear regression was formed for every possible ratio of large to small axon channel properties at each temperature. All ratios are shown as the larger axon property over the smaller axon property. (TIF) [file pcbi.1008057.s005.tif]
