# Supplementary material for: Temperature-robust activity patterns arise from coordinated axonal Sodium channel properties
Source: PLoS Comput Biol. 2020 Jul 27;16(7):e1008057. doi: 10.1371/journal.pcbi.1008057 (PMC7410338; doi:10.1371/journal.pcbi.1008057)
Supplement: Attachment — Submitted filename: plos review.pdf [file pcbi.1008057.s007.pdf]

In this manuscript, the authors utilize extracellular recordings, anatomical measurements, and a computational model to explore 1) the temperature dependence of action potential (spike) propagation speed, and 2) mechanisms that could reduce spike timing delays resulting from changes in temperature. This is a novel, careful study that extends recent important work that has focused on timing associated with spike generation. In the computational portion of the paper, the authors pose and largely answer the following questions:

- Which channel processes (Q10s) are important for determining the velocity Q10?
- Can different sets of channel Q10s combine to yield very similar spike velocity Q10s?
- Can channel properties be coordinated (and, if so, which ones?) to provide particular velocity Q10s for axons with very different diameters (and hence spike velocities), such that the spike delay is kept relatively constant as temperature is varied, and in these cases, how much do the "unimportant" properties matter?

My primary critique of this interesting paper is that the significance of the work could be made more accessible to the reader through clearer description of the analyses of the results of the computational model.

Major comments/suggestions

1) Experimental studies. I suggest that the authors use the experimental results as motivation for the computational work, rather than making too much effort to try to explain the mechanisms responsible for the experimental results themselves. The computational part of the project might be best seen as an exploration motivated by the experimental results.

   The authors' experimental results include the following: a) measurements of axon diameters for 3 pyloric neurons, b) measurements of the amplitude of extracellular recordings of spikes of those neurons made from the *dvn* and *lvn*, c) measurements of the three spike velocities, and d) measurements of the temperature dependence of the spike velocities. In particular, although there is considerable variation in the results of a-c, the temperature dependencies of the velocities (Q10s) for the three spikes were found to be very similar.

   In my opinion, the authors overemphasize the significance of the larger Q10 for PD (vs LP) compensating for its smaller spike velocity and hence minimizing effects on spike timing due to a change in temperature. This result should be reported, but I don't see it meriting the attention the authors pay to it for a number of reasons:

   a) Because PY has both the smallest spike velocity and Q10, its onset is delayed compared to PD. Why should preservation of the timing between LP and PD spikes be more important than that between PY and PD spikes?

   b) Regarding PY, however, the delay even at 25°C is still quite small - a few ms - a result of the Q10s of all three neurons being small (~1.5) and very similar.

c) Related to this point, I would suggest focusing on these very modest changes in timing, rather than on changes in the phases of the onsets of spiking. While maintenance of the phase of the spiking of the three neurons is important for the behavior (and STG people love to talk about phase), the effects on PY phase due to temperature are smaller than the variations observed across preparations and are also are comparable to calculated phase differences between the spiking measured on the *dvn* near the STG versus on the *lvn*. (An LP spike traveling at 90 cm/s takes 44 ms to travel 4 cm, while a PY spike traveling at 60 cm/s takes 67 ms, meaning there is a lag of 23 ms, approximately 2% of a typical pyloric period.) In any case, for the muscle movements controlled by these neurons, it would be hard to imagine that a phase change of 1-2% would matter for the behavior. Moreover, the more general audience for this paper likely is not as familiar with phase as with temporal delays. As such, I think Fig 2B could be eliminated or modified so that the vertical axis (or one of the vertical axes) would have units of time.

The authors might consider elaborating further on the following points:

d) The measured diameters of the LP and PD axons differed by approximately 5%, while the velocities differed by much more, as did the amplitudes of extracellular spikes measure on the *dvn* (Fig 1D, top). The HH result is that conduction velocity is proportional to the square root of diameter. Might this suggest that the axons have different sets of conductances? This would be interesting in that then there would be less reason to expect that the Q10s should be so similar.

e) The results of Fig. 2A are both expected and surprising. They are expected, because if there were some very dramatic effect on spike timing, the behavior itself would be affected within the animal's normal temperature range. They are surprising, or at least merit more discussion, for two reasons. First, the value of the velocity Q10s are themselves small, while it is known that the Q10s for channel processes can be much larger. Second, the three Q10s are very similar, despite evidence/suspicion that the conductances differ for the axons of the three neurons, and/or even for the same axon, preparation to preparation.

f) As an aside, the assignment of a specific value of Q10 for velocity is a little suspect, given the results of Fig 2A. For 5-15°C, the temperature dependencies look nearly identical; only above 15°C, do they diverge. The fits to lines in Fig 2A(iii) are ok, but not great.

2) <u>Computational studies (last section)</u>. I suggest that the authors edit and simplify the Results section entitled, "What channel properties need to be coordinated..." (p. 15), explain Fig. 4 more clearly, and refer carefully to the results and figure in the corresponding part of the discussion (p. 26-29). Here is where they draw their most important conclusion - that coordination of sodium channel properties across axons can result in robust

temperature-insensitive timing. This is summarized quite clearly in lines 436-446; however, the actual results are buried in a complicated description and analysis. I have read it a number of times, but I am still finding the section hard to follow. While I get the main points, I hope, I'm not sure I follow all the reasoning between lines 335 and 435. Some specific comments/suggestions follow:

a) Figure 4 is very complicated. I understand part A, although I think the caption could be explained in more detail. Line 300, sentence starting, "It is thus mostly the properties of the smaller axon…" Could this be explained in more detail? Is this a result of the range of velocity Q10 values of the small axon being different than that for the larger axon? Going from Q10s for channel properties to timing differences without commenting on the resulting velocity Q10s for the two axons makes it harder to gain intuition.

b) Figure 4B. Caption. What does "Example" mean in line 313? What is being held constant as the ratio of the particular Q10 is being changed for the bigger and smaller axon? All other Q10s?

c) Table 1 is overwhelming, and presumably only some of the entries are important for the discussion. How these numbers were obtained and what they mean should be explained in the table caption. I might consider moving this table to an appendix and referring only to a few entries in the text.

d) Line 354. How many models are there in total?

e) Line 361 and following. I suggest defining "necessity and sufficiency" more clearly. My understanding of "necessity" is that the two axons require a particular ratio to be within some range, and if it's not, then there won't be robust timing. "Sufficiency" means that if the particular ratio is within the range, the other ratios or actual Q10 values don't matter - the timing will be robust.

f) Lines 397-398. The authors looked at the ratios of the other Q10s and found that all ratios produced both robust and poor timing. But did they examine the ratios of the other Q10s for the subset of models in which the particular sodium channel ratios were constrained to the "good" range? I think this is what Fig. 4C (top) is getting at.

g) Line 549. Sentence beginning "Ultimately…" I think this is a key point that the authors are making, and I still cannot quite get it. Goes back to a need for clearer explanation in the Results. Related issue on line 562. Authors state sodium inactivation gate becomes more important, but in Fig. 4C, tau_h stays at the random chance level, regardless of the ratio.

Minor suggestions:

1. In a sentence or two, the authors should explain the source of the Q10s for the channel properties. I believe for the time constants / rates, these originate in the Arrhenius Equation / Boltzmann factor, and for the maximal conductances, this is due to diffusion. I have read that the Q10s for the former can be very large, but is it realistic to have Q10s of 3 or 4 for the maximal conductance, as the authors have done in their simulations? From https://link.springer.com/content/pdf/10.1007/978-1-4614-6675-8_236.pdf: *"The rate of opening and closing of ion channels typically has a Q10 in the range 2.4–4 (Hille 2001). The maximum conductance of an ion channel varies with temperature with a Q10 in the range 1.2–1.5 (Hodgkin et al. 1952; Hille 2001)."*

2. Figure 1Bi / fluorescence experiments. How many times was this experiment repeated? Do the authors expect (or have they observed) that the diameters vary preparation to preparation?

3. Figure 1G. At which position were the extracellular waveforms recorded? (I don't think it matters, but it should be reported.)

4. Line 140. How is the statement that axon diameters predict propagation velocities justified, mathematically? I agree that total current (or spike amplitude) is correlated with velocity. What does "predict" even mean in this sentence?

5. Lines 151-154. Related to prior comments. I agree that there is statistical significance. I question whether there is any physiological significance.

6. Lines 175-178. Sentence beginning "The reason…", I believe, is wrong. If the Q10s are the same, the two velocities will increase proportionally by the same factor, by definition. However, this does not equate to no change in timing.

7. Lines 179-182. See prior comments. I suggest restating this more simply saying that the relative values of the various Q10s are consistent both with the very small changes in timing between LP and PD and the larger change (and sign of the change) between PY and PD.

8. Line 214. Is there a diameter of this model axon? 3 μm? Lines 287 and following mention "two additional axons" (6 and 12 μm).

9. Line 223 / Fig. 3A. Holding everything else equal at canonical values?

10. Fig. 3C (right). Suggest removing the numbers on the vertical axis, or at least putting them in the correct positions. Is the range of the vertical axis the same as on Fig. 3C (left)? What about the models with Q10 values < 1? (Line 268)

11. Line 248. Why were the Q10s for the potassium channel set to 1.5? Is this because the velocity Q10s of pyloric axons were approximately 1.5? (Not sure there is any obvious connection.) Or because this is a reasonable value?

12. Line 256-259. The effect of the sodium inactivation gate Q10 on velocity in Fig. 3A is very modest. Is the fact that it can't be seen in Fig. 3B due to it no longer having a measurable effect or simply a function of the binning/color scheme?

13. Lines 523-528. Why is the low variability in velocity Q10 surprising? Do these channel proteins correlate to different Q10 channel values?

14. Line 531. Add "In the axons of three pyloric neurons…"

15. Line 543. "Data...demonstrate"

16. Line 548. Sentence starting, "The model now shows…" seems obvious, unless I'm missing something. This is known independently of any model.

17. Line 571-573. I don't think this statement is justified. The relatively precise timing is largely a function of all three Q10s being small.

18. The authors discuss correlated channel properties that compensate for changes in spike timing. Given the diversity seen neuron to neuron in the STG, which presumably could extend axon to axon, what mechanism(s) do the authors propose (if any) for correlating channel properties so as to minimize timing differences? Or might axons, in this respect, be less varied than the areas in the neuron where spikes are generated?
