# Supplementary material for: Temperature-robust activity patterns arise from coordinated axonal Sodium channel properties
Source: PLoS Comput Biol. 2020 Jul 27;16(7):e1008057. doi: 10.1371/journal.pcbi.1008057 (PMC7410338; doi:10.1371/journal.pcbi.1008057)
Supplement: Attachment — Submitted filename: Response to Reviewer comments.docx [file pcbi.1008057.s008.docx]

**Authors’ response:**

We would like to graciously thank the reviewers for the helpful critiques of our manuscript. Your suggestions have greatly improved cohesion between the experimental and computational results, and readability of the more complex components of the data. We have incorporated the reviewers’ suggested changes. Below, you will find our detailed responses to the reviewers’ comments highlighted in red following each comment.

All changes are also highlighted in red in the new manuscript.

**Reviewer 1:**

In this manuscript, the authors utilize extracellular recordings, anatomical measurements, and a computational model to explore 1) the temperature dependence of action potential (spike) propagation speed, and 2) mechanisms that could reduce spike timing delays resulting from changes in temperature. This is a novel, careful study that extends recent important work that has focused on timing associated with spike generation. In the computational portion of the paper, the authors pose and largely answer the following questions:

- Which channel processes (Q10s) are important for determining the velocity Q10?
- Can different sets of channel Q10s combine to yield very similar spike velocity Q10s?
- Can channel properties be coordinated (and, if so, which ones?) to provide particular velocity Q10s for axons with very different diameters (and hence spike velocities), such that the spike delay is kept relatively constant as temperature is varied, and in these cases, how much do the “unimportant” properties matter?

My primary critique of this interesting paper is that the significance of the work could be made more accessible to the reader through clearer description of the analyses of the results of the computational model. Major comments/suggestions

1) Experimental studies . I suggest that the authors use the experimental results as motivation for the computational work, rather than making too much effort to try to explain the mechanisms responsible for the experimental results themselves. The computational part of the project might be best seen as an exploration motivated by the experimental results. The authors' experimental results include the following: a) measurements of axon diameters for 3 pyloric neurons, b) measurements of the amplitude of extracellular recordings of spikes of those neurons made from the dvn and lvn , c) measurements of the three spike velocities, and d) measurements of the temperature dependence of the spike velocities. In particular, although there is considerable variation in the results of a-c, the temperature dependencies of the velocities (Q10s) for the three spikes were found to be very similar. In my opinion, the authors overemphasize the significance of the larger Q10 for PD (vs LP) compensating for its smaller spike velocity and hence minimizing effects on spike timing due to a change in temperature. This result should be reported, but I don’t see it meriting the attention the authors pay to it for a number of reasons:

a) Because PY has both the smallest spike velocity and Q10, its onset is delayed compared to PD. Why should preservation of the timing between LP and PD spikes be more important than that between PY and PD spikes?

b) Regarding PY, however, the delay even at 25°C is still quite small - a few ms - a result of the Q10s of all three neurons being small (~1.5) and very similar.

c) Related to this point, I would suggest focusing on these very modest changes in timing, rather than on changes in the phases of the onsets of spiking. While maintenance of the phase of the spiking of the three neurons is important for the behavior (and STG people love to talk about phase), the effects on PY phase due to temperature are smaller than the variations observed across preparations and are also are comparable to calculated phase differences between the spiking measured on the dvn near the STG versus on the lvn . (An LP spike traveling at 90 cm/s takes 44 ms to travel 4 cm, while a PY spike traveling at 60 cm/s takes 67 ms, meaning there is a lag of 23 ms, approximately 2% of a typical pyloric period.) In any case, for the muscle movements controlled by these neurons, it would be hard to imagine that a phase change of 1-2% would matter for the behavior. Moreover, the more general audience for this paper likely is not as familiar with phase as with temporal delays. As such, I think Fig 2B could be eliminated or modified so that the vertical axis (or one of the vertical axes) would have units of time.

Authors’ response:

Thank you for the very detailed and thoughtful comments. We appreciate the reviewer's suggestions and their constructive feedback. We believe that we have addressed all comments appropriately.

We have significantly revised the results sections pertaining to the motivation of the computational results to reflect the reviewer's comments to provide the experimental data as motivation for the modeling, and not to use the model to explain the biological mechanisms observed. We now refer to the biological data throughout model section to confirm that the model is predicting biologically relevant results. Additionally, we have revised the biological results text to better tie to decisions we made while generating the model. Specifically, the biological results text now emphasizes velocity differences, rather than diameter differences. This is echoed in the model description text in the results where we explain that the diameter differences are a means to achieve velocity differences in otherwise identical axons.

We also critically considered the reviewers criticism that we overemphasize the Q10 differences and their role in maintaining functional activity. We have revised the text to focus more on the generally low velocity Q10s of all three axons as support for temperature-robust activity. We have included a new Fig2b which shows the changes in arrival time, to accompany the changes in phase (now Fig 2C). We agree with the reviewer that the changes we observed are minute (<30ms) and while they may be statistically significant clearly do not disrupt the functional pattern and timing of the pyloric rhythm. We now state this more clearly in the text. Additionally, by emphasizing the similarity of low velocity Q10s in all three neurons, we also removed text suggesting that the LP-PD relationship would matter more than the PY-PD relationship.

The authors might consider elaborating further on the following points:

d) The measured diameters of the LP and PD axons differed by approximately 5%, while the velocities differed by much more, as did the amplitudes of extracellular spikes measure on the dvn (Fig 1D, top). The HH result is that conduction velocity is proportional to the square root of diameter. Might this suggest that the axons have different sets of conductances? This would be interesting in that then there would be less reason to expect that the Q10s should be so similar.

Authors’ response: We revised the results text to reflect the reviewer's comments by including additional comparisons of the measured axon diameters, waveform amplitudes, and velocity. This was primarily accomplished by reorganizing the section entitled "pyloric axons propagate APs at different velocities" to focus on the velocity differences first, and then using the amplitude and diameter measurement as support for the velocity differences. We completely agree that there is a mismatch between the waveform amplitude that was expected based on the axon diameter and what the measured velocities, and we have explicitly stated this now. Additionally, we have included a final paragraph which goes into more detail about the implications that the axonal conductances differ.

e) The results of Fig. 2A are both expected and surprising. They are expected, because if there were some very dramatic effect on spike timing, the behavior itself would be affected within the animal's normal temperature range. They are surprising, or at least merit more discussion, for two reasons. First, the value of the velocity Q10s are themselves small, while it is known that the Q10s for channel processes can be much larger. Second, the three Q10s are very similar, despite evidence/suspicion that the conductances differ for the axons of the three neurons, and/or even for the same axon, preparation to preparation.

Authors’ response: We agree with the reviewer’s assessment of figure 2 and have included more detail about its implications. Specifically, we provide more detail about the possibility of intrinsic differences in the axons to highlight our surprise at how similarly low their velocity Q10s were. At the same time, we condensed our description of the phase analysis (fig 2C) and added changes to arrival time (fig 2B) to highlight that the changes to timing are minimal, suggesting that the axons as a whole support temperature robust activity and behaviors.

Additionally, in the results of the computational model we refer to these biological results (figs 3B,C), adding some perspective to the possibility that the axons could have both distinct properties and also similarly low velocity Q10s.

f) As an aside, the assignment of a specific value of Q10 for velocity is a little suspect, given the results of Fig 2A. For 5-15°C, the temperature dependencies look nearly identical; only above 15°C, do they diverge. The fits to lines in Fig 2A(iii) are ok, but not great.

Authors’ response: We agree with the reviewer that the Q_10_ is likely not the ideal measurement for the data. Instead, the data has a more sigmoidal shape. We have now included in the supplemental a sigmoidal fit of the data, which provides a near perfect fit. The slope at the sigmoidal inflection point, matches the relative order of Q_10_ we have described, in that, PD has the largest slope, LP intermediate, and PY the smallest. Since these two fits complement one another, and Q_10_ is a standard measurement of temperature response, we have left in our description of the Q_10_ as well.

2) Computational studies (last section) . I suggest that the authors edit and simplify the Results section entitled, "What channel properties need to be coordinated..." (p. 15), explain Fig. 4 more clearly, and refer carefully to the results and figure in the corresponding part of the discussion (p. 26-29). Here is where they draw their most important conclusion - that coordination of sodium channel properties across axons can result in robust temperature-insensitive timing. This is summarized quite clearly in lines 436-446; however, the actual results are buried in a complicated description and analysis. I have read it a number of times, but I am still finding the section hard to follow. While I get the main points, I hope, I’m not sure I follow all the reasoning between lines 335 and 435. Some specific comments/suggestions follow:

a) Figure 4 is very complicated. I understand part A, although I think the caption could be explained in more detail. Line 300, sentence starting, “It is thus mostly the properties of the smaller axon…” Could this be explained in more detail? Is this a result of the range of velocity Q10 values of the small axon being different than that for the larger axon? Going from Q10s for channel properties to timing differences without commenting on the resulting velocity Q10s for the two axons makes it harder to gain intuition.

Authors’ response: We have included extensive new text and additional details in both the results text and in the figure legend to better explain Figure 4, and the main conclusions we are taking away from it. We have also edited the results text to clarify how we came to the conclusion that the properties of the smaller have a greater influences on the timing between the axons. In short, when the same range of parameters is altered in the smaller axon, it has a larger effect on the delay between the axons. This is now also stated in the text. Finally, the velocity Q_10_s for all axons are the same and correspond to those in Fig. 3. In short, modifying all the channel properties with the same Q_10_, resulted in the same velocity Q_10_ independent of the initial velocity/axon diameter. We added a sentence to state that the velocity Q_10_s are the same.

b) Figure 4B. Caption. What does “Example” mean in line 313? What is being held constant as the ratio of the particular Q10 is being changed for the bigger and smaller axon? All other Q10s?

Authors’ response: We have added more details to the results text to better explain Figure 4B. In short, nothing is being held constant – we plotted the delay associated with the ratio of two channel properties (here Sodium activation gate time constant) in both the 6µs and 3µm axon for every possible combination of other channel properties present. Because the total number of points plotted in greater than 65,000, we felt this distracted from the main purpose of the figure which is to show which ratios had a strong influence on timing. We thus opted to only show the linear regression, and included the R^2^ (a measure of how well the regression fit the data) in the supplementary table 1.

c) Table 1 is overwhelming, and presumably only some of the entries are important for the discussion. How these numbers were obtained and what they mean should be explained in the table caption. I might consider moving this table to an appendix and referring only to a few entries in the text.

Authors’ response: We have included a better description of how and why the fits we determined in the results text, and have made better reference to the ratios important for the key findings. We have moved the table into the supplementary data as Supplementary 3 Table.

d) Line 354. How many models are there in total?

Authors’ response: At each temperature there at 65, 536 models total. We have made reference to this in the text, and given the number of models associated with the percentages in the text.

e) Line 361 and following. I suggest defining “necessity and sufficiency” more clearly. My understanding of “necessity” is that the two axons require a particular ratio to be within some range, and if it’s not, then there won’t be robust timing. “Sufficiency” means that if the particular ratio is within the range, the other ratios or actual Q10 values don’t matter - the timing will be robust.

Authors’ response: Yes, our intention was to define these terms as the reviewer has described. We have clarified the definition of necessity and sufficiency in the text as the reviewer suggested.

f) Lines 397-398. The authors looked at the ratios of the other Q10s and found that all ratios produced both robust and poor timing. But did they examine the ratios of the other Q10s for the subset of models in which the particular sodium channel ratios were constrained to the “good” range? I think this is what Fig. 4C (top) is getting at.

Authors’ response: We did not examine whether other channel property ratios predicted delay for the subset of models that had “good” m/m or /gna ratios because they were already temperature robust models. We did however, examine the specific Q10s that were present for different ratios. While the “good” ratios were necessary for robust timing, they were not sufficient – meaning there were many models with a “good” ratio that were not temperature robust. Fig 4C shows that the ratio was insufficient because at some ratios, only particular Q10s were permissible to still result in temperature-robust timing. In other words, some Q10s were constrained, in addition to the 'good' ratio. We have provided more details in our description of the results text and figure legend pertaining to figure 4C.

g) Line 549. Sentence beginning “Ultimately…” I think this is a key point that the authors are making, and I still cannot quite get it. Goes back to a need for clearer explanation in the Results.

Authors’ response: This specific sentence no longer exists due to the extensive modifications of the text.

Related issue on line 562. Authors state sodium inactivation gate becomes more important, but in Fig. 4C, tau_h stays at the random chance level, regardless of the ratio.

Authors’ response: Yes, this is incorrect, we have removed tau h from the sentence.

Minor suggestions:

1. In a sentence or two, the authors should explain the source of the Q10s for the channel properties. I believe for the time constants / rates, these originate in the Arrhenius Equation / Boltzmann factor, and for the maximal conductances, this is due to diffusion. I have read that the Q10s for the former can be very large, but is it realistic to have Q10s of 3 or 4 for the maximal conductance, as the authors have done in their simulations? From https://link.springer.com/content/pdf/10.1007/978-1-4614-6675-8_236.pdf : “The rate of opening and closing of ion channels typically has a Q10 in the range 2.4–4 (Hille 2001). The maximum conductance of an ion channel varies with temperature with a Q10 in the range 1.2–1.5 (Hodgkin et al. 1952; Hille 2001). ”

Authors’ response: We have included a few sentences in the text describing our choices. In short, on top of what one usually defines as Q10 - the immediate response of the axon to temperature changes - our model includes effects on ion channels caused by extrinsic modulation or long-term homeostatic changes that have the ability to alter the temperature response of the axon. If temperature-dependent, these effects can be represented as a Q10. For example, an increased expression of ion channels would increase the maximum conductance in a temperature dependent way.

2. Figure 1Bi / fluorescence experiments. How many times was this experiment repeated? Do the authors expect (or have they observed) that the diameters vary preparation to preparation?

Authors’ response: The confocal experiment was not repeated, but we analyzed diameters using a regular fluorescent microscope. This is now stated in the results and methods. We have additionally shifted the focus of the results away from the (small) difference in diameter and towards the more prominent difference in velocity.

3. Figure 1G. At which position were the extracellular waveforms recorded? (I don’t think it matters, but it should be reported.)

Authors’ response: We have included this in the figure legend and results text.

4. Line 140. How is the statement that axon diameters predict propagation velocities justified, mathematically? I agree that total current (or spike amplitude) is correlated with velocity. What does “predict” even mean in this sentence?

Authors’ response: True, ‘predict’ should not be used here. We wanted to conclude that the expected relationship (larger axons = more current = faster propagation) was present. We have revised the manuscript to remove the word predict and stated the conclusion more simply.

5. Lines 151-154. Related to prior comments. I agree that there is statistical significance. I question whether there is any physiological significance.

Authors’ response: Agreed, we have revised the conclusion.

6. Lines 175-178. Sentence beginning “The reason…”, I believe, is wrong. If the Q10s are the same, the two velocities will increase proportionally by the same factor, by definition. However, this does not equate to no change in timing.

Authors’ response: Agreed, we have revised the sentence.

7. Lines 179-182. See prior comments. I suggest restating this more simply saying that the relative values of the various Q10s are consistent both with the very small changes in timing between LP and PD and the larger change (and sign of the change) between PY and PD.

Authors’ response: We have added this suggestion later, after the actual presentation of the results.

8. Line 214. Is there a diameter of this model axon? 3 μm? Lines 287 and following mention “two additional axons” (6 and 12 μm).

Authors’ response: yes, it is now included in the text.

9. Line 223 / Fig. 3A. Holding everything else equal at canonical values?

Authors’ response: yes, it is now included in the text.

10. Fig. 3C (right). Suggest removing the numbers on the vertical axis, or at least putting them in the correct positions. Is the range of the vertical axis the same as on Fig. 3C (left)? What about the models with Q10 values < 1? (Line 268)

Authors’ response: The data in Fig. 3C, right, are binned, so the numbers represent the middle of the bin and are in the right place. However, we have now included a better description of the data in figure 3C. With respect to Q10 values <1: In the range of temperatures measured (5-15°C), there were no velocity Q10s lower than 1. However, we recognize that this is not the case for all temperatures (there are some at 30C). To account for these models, we included Supplementary 4 Figure to show the velocity Q10 measured in 5 degree increments up through 30C. Overall this did not change the distribution of velocity Q10s.

11. Line 248. Why were the Q10s for the potassium channel set to 1.5? Is this because the velocity Q10s of pyloric axons were approximately 1.5? (Not sure there is any obvious connection.) Or because this is a reasonable value?

Authors’ response: The Q10 of the potassium channel were set to 1.5 to reflect literature values of the properties. We now state this also in the text.

12. Line 256-259. The effect of the sodium inactivation gate Q10 on velocity in Fig. 3A is very modest. Is the fact that it can’t be seen in Fig. 3B due to it no longer having a measurable effect or simply a function of the binning/color scheme?

Authors’ response: Correct, it was a function of the color scheme. The effect is measurable but basically an order of magnitude smaller than the influence of the sodium channels. We have edited the text to reflect this.

13. Lines 523-528. Why is the low variability in velocity Q10 surprising? Do these channel proteins correlate to different Q10 channel values?

Authors’ response: We found it surprising that such a large range of channel property Q10 values resulted in similar velocity Q10s. We have edited this section to make a better connection between the computational and experimental data.

14. Line 531. Add “In the axons of three pyloric neurons…”

Authors’ response: Thank you - changed as requested

15. Line 543. “Data...demonstrate”

Author response: Thank you - changed as requested

16. Line 548. Sentence starting, “The model now shows…” seems obvious, unless I’m missing something. This is known independently of any model.

Authors’ response: We have revised the statement.

17. Line 571-573. I don’t think this statement is justified. The relatively precise timing is largely a function of all three Q10s being small.

Authors’ response: We have removed this sentence and revised the paragraph to reflect the changes in focus of the results.

18. The authors discuss correlated channel properties that compensate for changes in spike timing.

Given the diversity seen neuron to neuron in the STG, which presumably could extend axon to axon, what mechanism(s) do the authors propose (if any) for correlating channel properties so as to minimize timing differences? Or might axons, in this respect, be less varied than the areas in the neuron where spikes are generated?

Authors’ response: There are several mechanisms that may contribute to regulating axonal temperature responses, including evolutionary, developmental and homeostatic pressures. Additionally, temperature-dependent neuromodulation may influence axon velocity. We felt though that speculating about mechanisms is beyond this manuscript as our experiments do not address this question and our model is not restricted to any given mechanism. We have, however, included a section at the end of the discussion where we mention homeostatic and long-term influences on the temperature response of axons. We additionally mention this in the results section when we introduce why the specific range of Q10 values for the different ion channel properties were chosen.

With respect to the idea that the axon properties vary less - this is possible although we are not aware of any publications that addresses this question.

**Reviewer 2:**

DeMaegd and Stein study how the pyloric rhythm produced by the stomatogastric ganglion can be preserved across a wide range of temperatures during transmission to muscles along axons. Using a preparation that allows measuring the conduction velocity in axons of different identified neurons at different temperatures, they find only mild temperature sensitivity of action potential velocity, resulting in a preserved phase relationship of the pyloric rhythm across temperatures. Additionally, they create a database of axon models with different temperature sensitivities of ion channels. Analysis of this database reveals relationships between parameters that allow preserving timing differences along axons across temperatures.
The authors perform nice experiments, but the finding that the pyloric rhythm is conserved in the axons across temperatures not very surprising. Further, the relationship between this main result and their modeling effort is not clear. Therefore, I cannot recommend this study for publication in its present form.

Major:

(1) The analysis of the relationship between different Q10s and timing differences between axons in Fig. 4 is nice, but the relationship to the experimental data is not clear. Is it possible to transform timing into phase differences and ask what combinations of Q10s result in phase differences within the experimentally determined range (see Fig. 2B)? If there is a large variety of Q10 combinations (even with some ‘coordination’) within this range of phase differences, this would be in line with ideas that in the CPG itself many different combinations of ion channel expression can result in a consistent pyloric rhythm – and that this principle also holds true for the transmission line to the periphery.

Authors’ response:

We appreciate the reviewer’s critical and thoughtful comments, we found them to be very helpful in improving our manuscript and constructively written.

We have edited this section of the results substantially. We have included a measurement of timing (Fig 2B) and provided a more detailed description of the phase analysis, and our reasoning for doing the phase analysis. We agree with the reviewer that there seems to be consistency with the principle that similar activity, or propagation, can be achieved through a large variety of channel combinations (though coordination may be beneficial for animals experiencing large environmental changes). We have highlighted this concept throughout the results text referring to the biological and computational data especially when considering figure 2B, 3B, and 4A.

(2) The model description is incomplete and some key assumptions are left unexplained. It is a compartmental HH-type model of a 4 cm long axon with a diameter of 3 µm (I assume from Fig. 4, but this isn’t mentioned anywhere). The number of compartments is not relevant, rather the length of an individual compartment (50 µm?). Also missing in the Methods is the value of the axial resistance.
More importantly, why were axon diameters of 3, 6 and 12 µm used instead of the measured diameters? This might help directly relating the simulation results to the experimental measurements (see above). Finally, it is an unusual idea to assume a temperature-dependence of the maximum conductance density. Since it is a model parameter, it certainly is fair game, however I am not aware that this is necessary for a HH description of Na and K channels. At the end of the discussion, the authors seem to imply that this is a phenomenological approximation of possible neuromodulatory influence on e.g. channel availability at different temperatures. If this is the case, please clarify this much earlier (and provide evidence if possible). Also, is an exponential temperature dependence an appropriate assumption in this case?
 Authors’ response:

We have included more details about the model in both the results and methods text, with specific reference to the details requested by the reviewer.

We chose axons of 3, 6 and 12µm diameter axons because our goal was to provide general features of axons of different velocities, and diameter differences are a means to achieve different velocities for a neuron with identical properties. We have now included this reasoning in the results text describing the model design. We use the biological data as a motivation for the computation approach, rather than using the computational approach to explain the details of our example biological system. We have tried to adjust the description of the model results to better reflect this.

With respect to our choices regarding the conductance Q10, we have followed the reviewer’s suggestion to clarify the possibility of neuromodulation we suggest in the discussion section. We have added several sentences in the description of the model in the section titled “Mechanisms underlying temperature-robust AP propagation,” about our reasoning for each Q10 range. In short, we wanted to include the effects of extrinsic modulation or long-term homeostatic changes in our description of the temperature response. For example, temperature-dependent extrinsic modulation could recruit additional channels, or influence the transcription of new channels and thereby increase the maximum conductance.

Minor:

-The temperature dependence of the conduction velocity does not seem to follow a single exponential (Fig. 2Ai and 2Aiii); there seem to be two different regimes (from 5-15 C and from 20-25 C). This poses a problem for the claim that the Q10 values are significantly different, since this assumes a single exponential. Is it possible to come up with a different phenomenological description (e.g., two Q10 values for the two ranges)? And do the axon models display this phenomenon, and could they be used to explain it?

Authors’ response: We have critically considered this comment and agree that a single exponential may not be the best description of the data. We have decided to maintain the descript of the Q10 because this is standard practice among literature describing temperature-dependency. However, we have also included an addition Supplemental figure showing that a Sigmoidal curve seems to precisely fit the data. The sigmoidal slope at inflection, and Q10 values indicate similarly that PD has the sharpest response to temperature, LP intermediate, and PY the smallest so we think including both is commentary. Additionally, very few axon models display this phenomenon, though we have included more discussion on those that do and included a supplemental figure on them as well. Briefly, we conclude that the reduced temperature response at high temperatures may be a result of pushing the axon towards its maximum temperature range (i.e. the temperature where it fails to generate action potentials).

-lines 127-128: The extracellular AP amplitude indeed seems to be characteristic for each axon, but that does not necessarily mean that the diameter difference is consistent everywhere (the amplitude could also be influenced by different ion channel densities).

Authors’ response: Agreed, we have significantly modified this section of the results to reflect the probable differences in intrinsic properties of the axons and focus more on the differences in velocity rather than diameter.

-lines 280-281: Unclear – maybe: ‘the temperature dependence of action conduction velocity is robust across a wide range of Q10s of the underlying ion channels’ or similar.
Authors’ response: We have incorporated the reviewer’s suggested simplification of the sentence into the manuscript.

-Please replace ‘preciseness’ by ‘precision’ throughout.

Author’s response: changed as requested.

-Fig. 4B: Please show examples of the data points with the linear regression to give an idea of how strong this relationship is

Authors’ response: We have decided not to include the data points of the linear regressions because it is a total of 65, 536 points for each slope shown. We felt that this over-encumbered the figure and detracted from the key finding that changes in the ratio between Sodium activation gate time constant Q10 of the smaller and larger axon caused the greatest changes in delay. However, we included the R^2^ values of the linear fits in the Supplementary 3 Table to indicate how well the linear regression fit the data, with larger R^2^ values indicating better fits.

-Switch the rows in Fig. 4C to match how they are referenced in the text

Authors’ response: We have changed the figure.

-lines 361-370: Please provide some form of significance test for these statements.

Authors’ response: We are unable to provide significance tests for these statements as these are the results of the model data. We have revised this section to explain in more detail how/why we have drawn the conclusions about the necessity and sufficiency of coordinating ion channel Q10 ratios for robust timing by incorporating not just the percentages of models, but the total numbers as well.

-lines 436-446: This is discussion, not a result.

Authors’ response: We left this section at the end of the results because we intended this as a summary and conclusion from the results, and another reviewer mentioned the utility of this section.

-lines 449-470: should not be part of discussion. Remove or integrate into introduction.

Authors’ response: We have integrated these paragraphs into the introduction.

-lines 546-547: Please clarify/remove. I don’t think Q10s of individual ion channels can be ‘regulated’ through gene expression.

Authors’ response: Agreed. We did not intend to imply this. We have removed the offensive text and revised the paragraph.

-lines 552-573: Seems very redundant with the results section.

Authors’ response: We have removed this sentence.

-line 704: I think the ln in the exponent needs to be a log10; i.e., R = Q10 ^ ((T-10)/10).

Authors’ response: Yes, that was a bad typo. Thank you for catching it.

**Reviewer 3:** This manuscript by DeMaegd & Stein addresses the question of the role of temperature in the conduction properties along the axon and activity patterns in the pyloric network of the crab stomatogastric system. In particular, the authors show that pyloric axons display variations in conduction velocity that are comparable over different types of axons (PD, LP, PY) yet displaying different conduction velocities and diameters. They next analyzed the reasons for this stability and found using a model that to maintain conduction timing similar amongst axon of different diameter, the sodium activation time constant (taum) and maximum sodium conductance (GNa) temperature sensitivity must be coordinated.
The results reported in this manuscript are interesting, the paper is well written but the presentation of some modeling results (see point 1 below) is hard to follow. In addition, the few points below should be addressed.
1. The color coding and data representation used in Figure 3B&C and 4A are really difficult to follow. The authors should consider a simplification of the message in both figures to allow the reader to easily extract pertinent information.

Authors’ response:

We thank the reviewer for their valuable comments and suggestions.

We have included significantly more detailed description of the figures in both the figure legends and results text to help the reader extract the pertinent information and focus on the key conclusions of the results.

2. It is concluded in the abstract and elsewhere in the paper that “the effects of temperature on action potential propagation were subtle”. This is not exactly true since in Figure 2A, the authors show a two-fold increase in velocity when the temperature goes from 5 to 20°C (i.e. Q10 ~1.5). Please, amend the conclusion in the abstract and throughout the paper accordingly.

Authors’ response: We have amended the conclusion in the abstract and rest of the manuscript as requested.

3. Figure 1B, the green axon has the same diameter as the red one. Perhaps, choose another region where the difference is not ambiguous.

Authors’ response: In response to comments from the other reviewers, we have revised the results substantially and included a better explanation for the small differences in diameter.

4. Please specify the used animal species (crab) in the abstract and in the results.

Author response: This has been changed.

5. Line 50, please insert a space (“, the” and not “,the”).

Authors’ response: Fixed

6. Page 18, line 355, add “between” after than.

Authors’ response: Changed

7. Page 19, line 375, 0.375 and not .375

Authors’ response: Changed as requested

8. Page 20, line 394, suppress the dot after “and”

Authors’ response: Changed as directed
